# Measuring the susceptibility to visually induced motion sickness and its relationship with vertigo, dizziness, migraine, syncope and personality traits

**DOI:** 10.1007/s00221-023-06603-y

**Published:** 2023-04-05

**Authors:** Ivana Lukacova, Behrang Keshavarz, John F. Golding

**Affiliations:** 1 Psychology, School for Social Sciences, University of Westminster, London, UK; 2 KITE-Toronto Rehabilitation Institute, University Health Network, Toronto, Canada; 3 Toronto Metropolitan University, Toronto, Canada

**Keywords:** VIMSSQ, MSSQ, Virtual Reality, Age, Sex, Survey, Vestibular, Persistent Postural-Perceptual Dizziness

## Abstract

The widespread use of visual technologies such as Virtual Reality increases the risk of visually induced motion sickness (VIMS). Previously, the 6-item short version of the Visually Induced Motion Sickness Susceptibility Questionnaire (VIMSSQ short form) has been validated for predicting individual variation in VIMS. The aim of the current study was to investigate how the susceptibility to VIMS is correlated with other relevant factors in the general population. A total of 440 participants (201 M, 239F), mean age 33.6 (SD 14.8) years, completed an anonymous online survey of various questionnaires including the VIMSSQ, Motion Sickness Susceptibility Questionnaire (MSSQ), Vertigo in City questionnaire (VIC), Migraine (scale), Social & Work Impact of Dizziness (SWID), Syncope (faintness), and Personality (‘Big Five’ TIPI). The VIMSSQ correlated positively with the MSSQ (*r* = 0.50), VIC (*r* = 0.45), Migraine (*r* = 0.44), SWID (*r* = 0.28), and Syncope (*r* = 0.15). The most efficient Multiple Linear Regression model for the VIMSSQ included the predictors MSSQ, Migraine, VIC, and Age and explained 40% of the variance. Factor analysis of strongest correlates with VIMSSQ revealed a single factor loading with VIMSSQ, MSSQ, VIC, Migraine, SWID, and Syncope, suggesting a common latent variable of sensitivity. The set of predictors for the VIMSSQ in the general population has similarity with those often observed in patients with vestibular disorders. Based on these correlational results, we suggest the existence of continuum of underlying risk factors for sensitivity, from healthy population to patients with extreme visual vertigo and perhaps Persistent Postural-Perceptual Dizziness.

## Introduction

Motion sickness is a common phenomenon resulting in various symptoms such as nausea, vomiting, headache, pallor, sweating, or drowsiness (see Keshavarz and Golding 2022; Lawson 2014; for overviews). Similarly, the use of visual technologies and devices such as Virtual Reality, smartphones, or video games can elicit motion sickness-like sensations, often referred to as visually induced motion sickness (VIMS) (Cha et al 2021). In these cases, physical motion cues are typically absent or limited and symptoms are primarily driven by stimulation of the visual system (Keshavarz et al. 2014). While classic motion sickness produced by transportation and VIMS share the same core symptomatology of gastrointestinal and autonomic symptoms, the occurrences of oculomotor and central symptoms such as eyestrain, dizziness and headache are relatively higher in VIMS (Cha et al 2021).

**Fig. 1:**
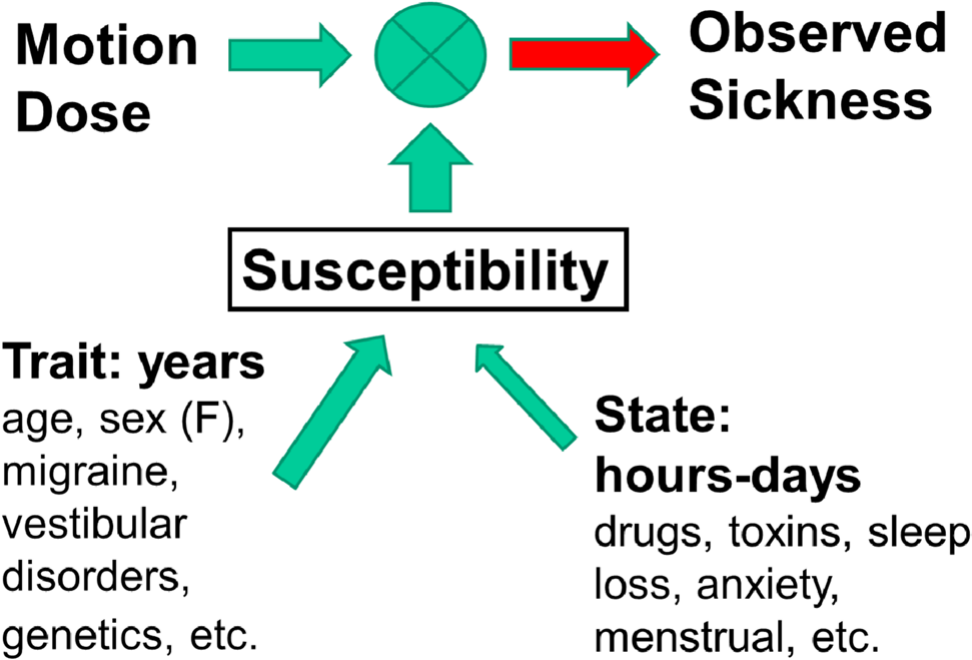
Schematic diagram illustrating the influences of State and Trait factors in determining individual susceptibility to motion sickness and VIMS. See text for details

Both classic motion sickness (produced by physical motion) and VIMS can be considered the product of the interaction between the strength of the provocative environment (sensory conflict potential, stimulus intensity, exposure duration) and an individual’s susceptibility to motion sickness and VIMS. Consequently, incidence rates of motion sickness and VIMS vary widely due to differences in susceptibility, which can be broadly divided as arising from two sources: individual state and trait characteristics (see Fig. 1).

State factors influencing motion sickness/VIMS vary over time periods of hours to days. Examples of state factors which can influence motion sickness susceptibility include anti-motion sickness drugs (Wood et al. 1969), emetic toxins (Yates et al 2014), immediate anxiety or fear (Besnard et al 2021), sleep loss (Kaplan et al 2017), female menstrual cycle (Golding et al 2005), and adaptation/habituation. However, with regards to the latter, habituation can acquire the properties of a trait if retained over a prolonged term (Benson 2002). A useful overall term denoting the influence of state factors on motion sickness susceptibility is the “ Dynamic Threshold for Nausea “, first coined by Stern (2002).

In contrast to state factors, trait factors are enduring or vary slowly over the years, including genetics/heritability, medical conditions (migraine, vestibular disorders), biological sex, age, or personality traits. In the past, the relationship between these trait factors and classic motion sickness has been well investigated. With regards to genetics, for instance, monozygotic versus dizygotic twin studies suggested that a large proportion of variation in susceptibility to motion sickness is accounted for by genetic factors, with heritability estimates of 55–70% (Reavley et al 2006). In addition, multiple genes appear involved and 35 single-nucleotide polymorphisms associated with motion sickness susceptibility have been identified (Hromatka et al 2015). Vestibular disorders may decrease or increase the risk of motion sickness. On the one hand, a complete, bilateral loss of labyrinthine function is thought to confer immunity to motion sickness (Kennedy et al. 1968; Cheung et al. 1991). However, a very small minority of bilateral labyrinthine defective individuals may be still susceptible to motion sickness provoked by visual stimuli designed to induce self-motion (vection) during pseudo-Coriolis stimulation (Johnson et al 1999). On the other hand, patients with vestibular pathology and vertigo such as Meniere’s disease or vestibular migraine are especially susceptible to motion sickness (Bronstein et al 2020). Several other pre-existing medical conditions are associated with raised motion sickness susceptibility, including dizziness (Bronstein et al 2010; Golding and Patel 2017) and proneness to Syncope (i.e. feeling of faintness; Bosser et al 2006). The influence of personality factors such as trait anxiety or neuroticism in raising susceptibility appears relatively weak (Reason and Brand 1975). Age and sex, since they are easy to measure, have been studied extensively in relation to motion sickness susceptibility. Age is an important factor, with infants and very young children being relatively immune to motion sickness (Reason and Brand 1975) and susceptibility peaking around 9 to 10 years (Turner and Griffin 1999). There is a subsequent decline of susceptibility during the teenage years towards adulthood around 20 years, probably due to habituation. Biological sex appears to play a role as well, with women somewhat more susceptible to motion sickness than men (Kennedy et al 1995). This is a much weaker effect than age and, generally, a less consistent finding (see reviews in Lawson 2014; Lawson et al 2021). However, any increased susceptibility is likely to be objective and not subjective because women also vomit more than men as a response to motion stimuli; surveys of passengers at sea indicate a 5 to 3 female to male risk ratio for vomiting (Lawther and Griffin 1986; 1988).

With regards to VIMS, the roles of biological sex and age have been frequently investigated. For instance, women have sometimes (D’Amour et al. 2017; Klosterhalfen et al. 2006; Flanagan et al. 2005) but not always (Stanney et al. 2020) been found to be more susceptible to visual stimulation than men. The situation may be complicated since it has been suggested that sex differences in susceptibility to VIMS may be stimulus specific, for example, VIMS might be less strong for rotary motion stimuli, but stronger for linear motion stimuli, but this remains unproven (Koslucher et al 2015). In contrast to classic motion sickness, children aged 4–10 seem less prone to VIMS (Chang et al. 2021), but an increase in susceptibility has been reported later in life with older adults (Keshavarz et al. 2018; Brooks et al. 2010), although this remains to be definitely proven. However, outside of biological sex and age, only little is known about the relationship between other trait characteristics and VIMS. Migraineurs (non-vestibular migraine) have been found to be more susceptible to VIMS provoked by visual stimuli (Drummond 2005; Golding and Patel 2017), but our knowledge on the influence of other trait characteristics on VIMS susceptibility remains weak. In a recent online survey, Keshavarz et al. (2021) correlated VIMS susceptibility measured via the long version of the VIMSSQ with dizziness and migraine susceptibility and observed correlations suggesting that those who tend to be more prone to dizziness and migraines also have a higher susceptibility to VIMS. However, it is not well understood how personality traits (e.g. extraversion, agreeableness) may be related to an individual’s susceptibility to VIMS.

To address this lack of knowledge, we conducted an online survey and measured trait characteristics (vestibular disorders, dizziness, migraine, syncope, personality traits, biological sex and age) as well as participants’ VIMS and motion sickness susceptibility using validated questionnaires. That is, we applied the Motion Sickness Susceptibility Questionnaires (MSSQ, sometimes called Motion History Questionnaires), a well-validated tool that enables a quick estimate of an individual’s susceptibility to classic motion sickness (Golding 2006). Importantly, the MSSQ was developed mainly to predict the risk of motion sickness to real (e.g. translational motion, cross-coupled motion, seasickness, airsickness) but not apparent motion. In the years subsequent to its development, the importance of visual technologies as a source of motion sickness has grown considerably. Therefore, recent work was undertaken to develop a questionnaire equivalent to the MSSQ specifically designed to improve the predictive power for VIMS. As a result, the Visually Induced Motion Sickness Susceptibility Questionnaire (VIMSSQ) was introduced (Keshavarz et al. 2019). The original version of the VIMSSQ is 67 items long and normative data from a large sample as well as supporting evidence from an experimental study for the predictive power of the original VIMSSQ have been previously reported (Keshavarz et al 2021). The original VIMSSQ was reduced to a short form consisting of only 6 items (Golding and Kesharvaz 2017; Golding et al. 2021), providing a quick and easy-to-apply tool for predicting VIMS. The predictive validity of the short form of the VIMSSQ for VIMS elicited by a standardised moving visual stimulus has been proven in the laboratory (Golding et al 2021). (*NB* for brevity in this paper we use the general term VIMSSQ, referring to the short form). However, data for the VIMSSQ from a large population sample are currently missing.

The current study had two main goals. First, we aimed to further investigate the relationship between VIMS susceptibility and relevant trait characteristics (migraine, dizziness, personality traits) to gain more insights into the relationship between these concepts. Second, by conducting a large-scale online survey, we desired to collect VIMSSQ data from a larger sample size that provides first insights on the distribution of VIMS symptoms in a broader population. However, as we did not systematically focus on age, ethnicity, or racial group, the data collected here offer limited normative data that need to be interpreted with care.

## Methods

### Participants

A total of 440 participants (201 males, 239 females) with a mean age of 33.6 years (SD = 14.8; age range 18 to 81) completed an anonymous online survey. Participants were fully briefed, gave informed consent, and were free to withdraw from the online survey at any time. Ethical approval was granted by the Psychology Ethics Committee of the University of Westminster, London UK (Ethics Approval for Research Based Project Application Registration ID: ETH1920-1402).

### Design

The study consisted of a cross-sectional design using an anonymous online survey (delivered via Qualtrics). Anonymous responding was employed since it encourages truthful self-reporting. Participants completed a battery of questionnaires (see below). Participants were recruited using a mixture of opportunity sampling including snowball sampling. The study was advertised online using social media (Instagram, Facebook, Twitter).

### Questionnaires

A variety of different questionnaires were administered to investigate their relationship with (and their efficacy for predicting) VIMS susceptibility as assessed by the VIMSSQ.

- *VIMSSQ.* The short form of the Visually Induced Motion Sickness Susceptibility Questionnaire VIMSSQ–a 6-item short version of the VIMSSQ (Golding and Keshavarz 2017; Golding et al 2021)–was developed to capture individual susceptibility to VIMS and was designed with the expectancy that it would be used in conjunction with the MSSQ as a supplement for circumstances when VIMS is anticipated. The VIMSSQ-short enquires about the frequency of 5 different symptoms (nausea, headache, fatigue, dizziness, eye strain) and also possible consequent avoidance when using a variety of visual devices and displays (e.g. smartphone, movie theatre, video games, tablets, Virtual Reality glasses, etc.). Items are scored 0 (never) to 3 (often). A total score is formed by the addition of all items giving a maximum possible range for the VIMSSQ total score of minimum of 0 to maximum of 18. Higher scores indicate a stronger susceptibility to VIMS. The VIMSSQ is shown in Table 1.
- *MSSQ.* The short form of the Motion Sickness Susceptibility Questionnaire MSSQ (Golding 2006) was used to assess the participants’ susceptibility to classic motion sickness from physical motion. The MSSQ enquires about the participants’ previous experiences of motion sickness when using 9 different modes of transportation (e.g. boat, car, bus, plane) or amusement rides (e.g. funfair rides). Participants rated the frequency of experiencing motion sickness for each item on a scale from 0 (never) to 3 (often). They could also indicate if they never used or experienced the respective item. The MSSQ has two sections, one asking about childhood experiences before the age of 12 (MSSQ Child) and one asking about experiences during adulthood over the last 10 years (MSSQ Adult). A raw score of the whole MSSQ scale can be calculated and, if required, can be translated into percentile scores based on the population norms reported in Golding (2006). Higher scores indicate a stronger susceptibility to motion sickness.
- *VIC.* The Vertigo in the City Questionnaire (VIC) (Golding 2015) is a short 5-tem scale, which was developed as part of a larger multi-disciplinary project to investigate common experiences of dizziness and vertigo experienced by people living in the urban or built environment. The VIC was subsequently tested and validated in a wider survey (Peverall and Golding 2017). The VIC does not mention motion sickness per se, and only enquires about experiences of vertigo/dizziness. The VIC asks respondents about their everyday experiences of dizziness and vertigo caused by visually stimulating devices and environments, such as moving display screens (e.g. advertising screens, information screens at tube stations, shopping malls), travelling on escalators, glass stairways, etc. Responses are rated on a binary scale (0 = *no*, 1 = *yes*). A summed total score (max. score = 5) can be produced in which higher scores indicate greater experiences of dizziness and/or vertigo in the urban and built environment.
- *Migraine Screen Questionnaire.* The Migraine Screen Questionnaire (Lainez et al. 2010) consists of five items that are rated on a binary scale (0 = *no*, 1 = *yes*) to measure the participants’ tendency to experience migraines. Items include, for instance, the person’s experience of frequent or intense headaches and the duration of those. A total score can be calculated by summing together the value of each item (max. score = 5). Higher scores indicate a greater likelihood of migraines.
- *SWID.* The Social Life and Work Impact of Dizziness questionnaire (SWID) measures the negative impact of dizziness on everyday activities (Bronstein et al 2010). The SWID consists of a set of four social, travel, family, and work-related questions, and has been validated in patient and control samples. Responses are rated on a binary scale (0 = *no*, 1 = *yes*) and a summed total score (max. score = 4). Again, higher scores indicate greater probability of being affected by dizziness.
- *Syncope.* A single item Syncope question was added to measure the participants’ tendency to experience vasovagal syncopes (Golding and Patel 2017). Participants had to indicate how often they experience the feeling of faintness (e.g. if stressed, in pain, or sighting blood), with higher scores indicates more frequent syncope. This single item question was adapted from Bosser et al. (2006).
- *TIPI.* The Ten Item Personality Inventory (TIPI) (Gosling et al. 2003) is a brief measure of the Big Five Personality Factors and was used to investigate the relationship between the personality factors extraversion, agreeableness, conscientiousness, emotional stability, and openness to experience. Participants rate their level of agreement with 10 statements (e.g. I see myself as extraverted/enthusiastic) on a scale from 1 (strongly disagree) to 7 (strongly agree) with balanced reverse scored items for each personality factor.

**Table 1:** Visually Induced Motion Sickness Susceptibility questionnaire short form version (VIMSSQ)

This questionnaire is designed to measure your experience with different visual display or entertainment devices and if they ever caused discomfort				
Visual display or entertainment devices include Movie Theatre or Cinema, Smartphones & Tablets with movies or games, Video games, Virtual Reality Glasses or Head Mounted Displays, Simulators, Large Public Moving Display Advertising or Information Screens				
Please answer these questions solely with respect to your experiences during adulthood (older than 18 years) and ignore childhood experiences				
Q1. How often have you experienced each of the following symptoms when using any of these devices? (circle your response)				
Nausea	Never	Rarely	Sometimes	Often
Headache	Never	Rarely	Sometimes	Often
Dizziness	Never	Rarely	Sometimes	Often
Fatigue	Never	Rarely	Sometimes	Often
Eye-Strain	Never	Rarely	Sometimes	Often
Q2. Have any of these symptoms stopped you using any of these devices or made you avoid viewing such displays? (circle your response)				
	Never	Rarely	Sometimes	Often
Q3. If you have answered stopped or avoided, please list the devices or displays that you avoid				

### Statistical analysis

Results were analysed using SPSS 27.0 (IBM^®^). Descriptives, correlations (Pearson and nonparametric), exploratory factor analysis, and multiple linear regression were employed. For all statistical analyses, the significance level was set to *α* = 0.05. Where statistical tests could be directional, the significances were 2-tailed.

## Results

### General descriptives

**Fig. 2:**
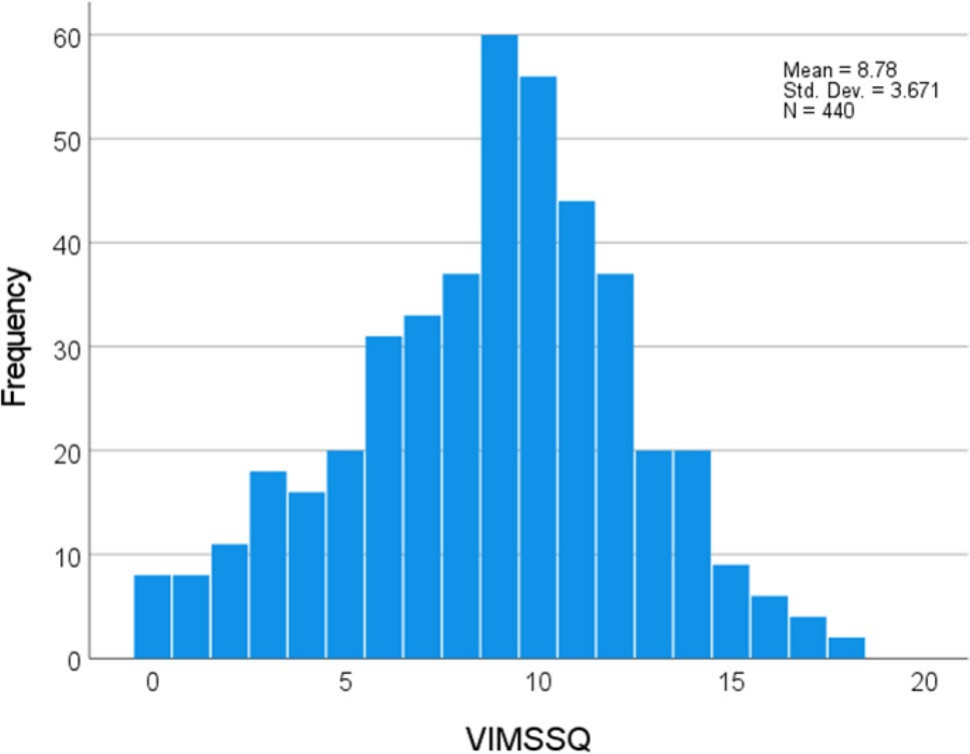
Distribution of the Visually Induced Motion Sickness Susceptibility Questionnaire (VIMSSQ) total score

Descriptives for all the questionnaires are given in Table 2, and detailed item breakdowns for the VIMSSQ in Table 3. The distribution of the VIMSSQ score is given in Fig. 2. Since the VIMSSQ is a relatively new scale, no published norms are available to compare with these data. However, the MSSQ has a norm of 12.9 (SD 9.9) (Golding 2006) which is lower than observed here (see Table 2), suggesting that this sample had a greater proportion of more motion susceptible individuals. This was confirmed by calculating the percentile scores of the MSSQ in these data, giving a mean percentile score of 75.47 (SD 25.01), which is significantly higher than the expected percentile norm which is 50 by definition (1-sample *t* test, *t*(438) = 21.3, *p* < 0.001, 2-tailed).

**Table 2:** Descriptives of all of the variables (*n* = 440)

Variable	Mean (SD) or %
VIMSSQ (Visually induced motion sickness) total score	8.78 (3.67)
MSSQ (Motion sickness susceptibility) score	23.22 (11.35)
VIC (Vertigo in the city) score	2.18 (1.58)
Migraine (Migraine screen) score	2.56 (1.50)
SWID (Social & work impact of dizziness) score	1.19 (1.32)
Syncope (Syncope experience) percentage	43%
Extraversion (Personality TIPI) score	4.32 (1.39)
Agreeableness (Personality TIPI) score	4.81 (1.09)
Conscientiousness (Personality TIPI) score	4.66 (1.42)
Emotional stability (Personality TIPI) score	4.25 (1.36)
Openness (Personality TIPI) score	4.62 (1.21)

**Table 3:** Breakdown of the VIMSSQ by question item, biological sex, and correlation of each item with the VIMSSQ total score

Item	Males *n* = 201		Females *n* = 239		Male + Female *n* = 440		Item correlation with VIMSSQ total score
	Mean	SD	Mean	SD	Mean	SD	*r*
Nausea	1.73	1.03	1.36	0.89	1.53	0.97	0.66
Headache	1.58	1.0	1.68	0.88	1.63	0.94	0.65
Dizziness	1.32	0.98	1.09	0.87	1.19	0.93	0.61
Fatigue	1.41	1.01	1.29	1.03	1.34	1.02	0.60
Eye-Strain	1.76	1,00	1.73	0.97	1.74	0.98	0.58
Avoidance	1.48	0.98	1.23	0.92	1.34	0.96	0.70
VIMSSQ total	9.27	4.06	8.36	3.25	8.78	3.67	1.00

As an indication of consistency of responding within the sample, it can be noted that the correlation of the MSSQ childhood subscale with adulthood subscale was *r* = 0.739 (*p* < 0.001). This was as good or exceeded the published normative correlation between these subscales of *r* = 0.68 (Golding 2006). On this basis, we can have some confidence in the consistency of responding within this sample.

### Correlations between variables

**Fig. 3:**
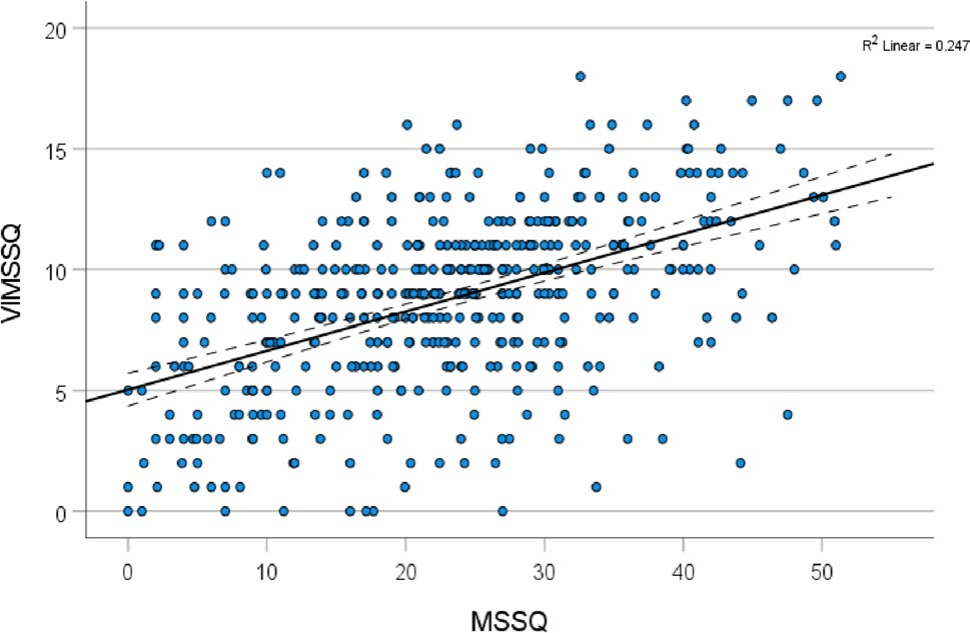
A scatterplot of the relationship between the Visually Induced Motion Sickness Susceptibility Questionnaire (VIMSSQ) total score and the Motion Sickness Susceptibility Questionnaire (MSSQ) score. The dotted lines represent the 95% CIs shown on either side of the fitted regression line. Each point represents an individual person, some points may overlap and represent more than one individual

Correlations (parametric Pearson r) between the VIMSSQ total score and the other variables are shown in Table 4. Additionally, nonparametric (Spearman) correlations are shown as an internal consistency check (Siegel 1956), suggesting that both parametric and nonparametric correlations revealed very similar results. Nonparametric statistics are by definition ‘distribution-free’ (Siegel 1956) and this check confirmed that the patterns of observed relationships were internally consistent and reliable. The strongest relationship with the VIMSSQ was found for the MSSQ, followed by lesser associations with VIC, Migraine, and SWID. The relationship between the VIMSSQ and the MSSQ is plotted in Fig. 3.

**Table 4:** Bivariate Correlations (*r*) of the VIMSSQ total score with other variables

Variable	*r*	*p*	(Spearman *r*)
MSSQ (Motion sickness susceptibility) total score	0.50	***	(0.48***)
VIC (Vertigo in the city) score	0.45	***	(0.43***)
Migraine (Migraine screen) score	0.44	***	(0.42***)
SWID (Social & work impact of dizziness) score	0.28	***	(0.29***)
Syncope (Syncope experience) percentage	0.15	**	(0.15**)
Extraversion (Personality TIPI) score	− 0.04	ns	(− 0.02 ns)
Agreeableness (Personality TIPI) score	− 0.14	**	(− 0.12*)
Conscientiousness (Personality TIPI) score	− 0.12	*	(− 0.11*)
Emotional stability (Personality TIPI) score	− 0.06	ns	(− 0.04 ns)
Openness (Personality TIPI) score	− 0.14	**	(− 0.12*)
Age	− 13	**	(− 0.12*)
Biological sex	− 12	**	(− 0.11*)

***p < .001, **p < .01, * *p* < .05,

*ns* not significants

The relationship between the VIMSSQ and the MSSQ (Fig. 3) can be used to estimate a corrected value of the VIMSSQ, based on the assumption that this sample was more motion sickness susceptible overall. The fitted regression line is: VIMSSQ-short = MSSQ × 0.16 + 5.04. Insertion of the expected MSSQ norm of 12.9 (Golding 2006), produced a corrected mean VIMSSQ of 7.10. The equivalent procedure using percentile converted MSSQ scores produced regression line equation VIMSSQ = MSSQ percentile × 0.07 + 3.46. Insertion of the expected MSSQ percentile norm of 50, providing a corrected mean VIMSSQ of 6.96. These corrected mean VIMSSQ values of 7.10 and 6.96 were very similar. Therefore, a good estimate of a norm for the VIMSSQ estimated on this basis, would be a mean score of 7.0.

### Multivariate analyses

**Fig. 4:**
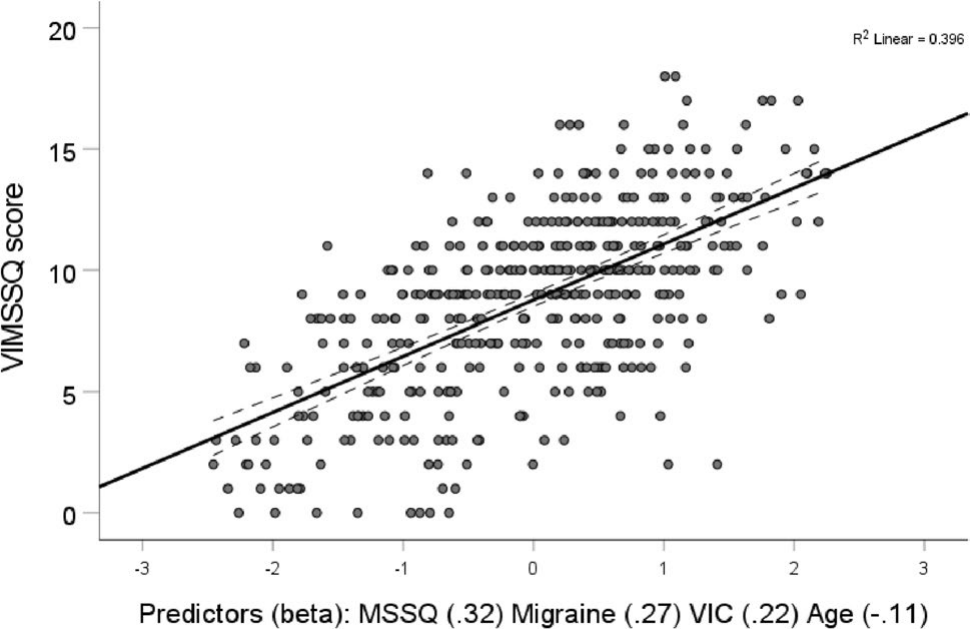
Multiple Linear Regression prediction of susceptibility to visually induced motion sickness measured by the VIMSSQ using the predictors MSSQ, Migraine, VIC, an Age. The standardised predictor is shown on the x-axis, with the beta values of the individual predictors. Dotted lines represent the 95%CIs shown on either side of the fitted regression line. Each point represents an individual person, some points may overlap and represent more than one individual

A number of multiple linear regression models, using both general and stepwise approaches, were explored to identify those variables best able to predict VIMS as measured by the VIMSSQ. The most efficient model (adjusted *R*^2^ = 0.4; *F*(4, 433) = 70.9, *p* < 0.001) included the predictors (beta, significance) MSSQ (0.32, *p* < 0.001), Migraine (0.27, *p* < 0.001), VIC (0.22, *p* < 0.001), and Age (− 0.11, *p* < 0.01) (see Fig. 4). The variables SWID, Syncope, Sex, and Personality traits were not included in this model. Perusal of the full correlation matrix indicated that this was due to either multi-collinearity (e.g. SWID & Syncope with the other predictors) or mundanely because their relationships with VIMSSQ were low or not significant (Sex, Personality). However, Age did not drop out from the final model, indicating that it did contribute significant unique predictive power, despite showing relatively weak bivariate correlation with the VIMSSQ.

Examination of the full correlation matrix showed that many of the variables correlated significantly with each other. A number of Exploratory Factor Analyses (for brevity not shown in detail here) were performed, with Varimax rotation if more than one component was revealed. Results suggested that, after rotation, the variables Personality Traits, Age, and Sex formed a single factor separate from the variables VIMSSQ, MSSQ, SWID, Migraine, VIC, and Syncope, which loaded on another main factor. A final Factor Analysis using the latter variables revealed a single factor loading, which had been previously identified as the strongest correlates with VIMSSQ (see earlier Table 4). This suggested a common latent variable of sensitivity. The results of this factor analysis are shown in Table 5.

**Table 5:** Factor analysis of the most important variables associated with the VIMSSQ

Variable	Loading
VIMSSQ (Visually Induced Motion Sickness)	0.718
MSSQ (Motion sickness susceptibility)	0.728
VIC (Vertigo in the city)	0.784
Migraine (Migraine screen)	0.627
SWID (Social & work impact of dizziness)	0.696
Syncope (Syncope experience)	0.486

Extraction method principal component analysis. One component extracted

Factor analysis extracted only a single component, suggesting a common latent variable of sensitivity (*N* = 440, 46.2% of variance)

## Discussion

The two main aims of the present study were to investigate factors that are related to VIMS susceptibility and to provide an estimate of the susceptibility to VIMS (and a distribution of the VIMSSQ) in the general population with a large sample. Greater susceptibility to VIMS was associated with greater susceptibility to classic motion sickness provoked by physical motion (MSSQ), increased experiences of dizziness and/or vertigo in the urban built environment (VIC), greater likelihood of migraine (Migraine Screen), greater experience of negative impacts of dizziness in social & work environments (SWID), and greater probability of experiencing vasovagal symptoms (Syncope). Weaker associations with the VIMS were found for age, sex, and personality traits (‘Big Five’ TIPI). We will discuss these findings in more detail in the following sections.

### VIMS, classic motion sickness, dizziness, and migraine

The strong correlation between the VIMSSQ and the MSSQ was to be expected, since VIMS and classic motion sickness share the same core symptomatology of gastrointestinal and autonomic responses (see Cha et al 2021). However, although highly significant, the correlations also indicated that there was much unshared variation and that the susceptibility to VIMS does not fully overlap with the susceptibility to classic motion sickness, making them similar but not identical phenomena (Keshavarz and Golding 2022). Similarly, the moderately strong correlations between the VIMSSQ and vertigo (as measured by the VIC) and dizziness (SWID) can be explained by the general importance of symptoms such as vertigo and dizziness for VIMS (Keshavarz et al. 2014). The link between VIMS, MSSQ, and dizziness has also been shown in previous experimental work. For instance, Golding et al. (2021) exposed participants to a tilted panoramic scene presented on a large screen that was constantly rotating along the vertical axis. Strong, significant correlations between VIMS as measured by the Simulator Sickness Questionnaire (Kennedy et al. 1993) and the MSSQ and SWID supported the idea that these concepts are closely related to each other.

Associations between migraine susceptibility with both VIMS and classic motion sickness have long been noted (Grunfeld and Gresty 1998; Golding and Patel 2017; Abouzari et al 2020). The results of this study were in accordance with these previous findings and suggest that VIMS, classic motion sickness, and the tendency to experience migraines are positively linked with each other. The mechanism underlying this relationship remains unknown; various suggestions include that it may be due to altered serotonergic system functioning or alternatively defective functioning of calcium ion channels (Golding 2016). Regardless of the exact mechanism, migraine appears to share underlying genetic factors with motion sickness susceptibility (Hromatka et al 2015). The significant association of syncope susceptibility with VIMSSQ supports the notion that autonomic reactivity may be an additional factor in motion sickness susceptibility, an observation consistent with previous findings with motion sickness both from real motion sources (Bosser et al 2006) and when provoked by visual stimuli (Golding et al 2021).

The overall relationship between the susceptibility to VIMS and the susceptibility to classic motion sickness with vertigo, migraine, and syncope appears consistent with the previous literature. However, much of the previous data on such relationships has concentrated on classic motion sickness with less information concerning VIMS. Moreover, with the exception of one previous study (Golding et al 2021), which had the limitation of a small sample, few if any previous studies have investigated VIMS susceptibility with all these variables simultaneously in a large sample. The importance of investigating all these variables at the same time is that it enables an estimate to be made of their relative importance in the same individuals.

### The relationship between VIMS, age, sex, and personality traits

Age, sex, and personality had lower correlations with VIMS susceptibility. There was a small decline in susceptibility to VIMS with increasing age. Similarly, age was retained as one of predictive factors in the multiple linear regression (together with MSSQ, Migraine, and VIC), suggesting that age does contribute some unique predictive power. This finding is consistent with the general literature concerning motion sickness susceptibility (Turner and Griffin 1999). No second peak of VIMS susceptibility could be identified in older ages. Although this study failed to support the idea that such a peak exists for VIMS in particular (Keshavarz et al. 2018; Brooks et al. 2010), our study despite having a wide age range, was not optimised to investigate effects in older age groups per se, and may have lacked power to detect hypothetical small peaks at older ages > 65 years. It is worth noting that the relationship of VIMS susceptibility with age over the adult years may be complicated (Golding et al 2021). People become more visually dependent with increasing age as they reweight the three main sensory inputs used for balance and orientation. The reweighting is usually away from vestibular and proprioceptive inputs (which often become less reliable with ageing) to greater dependence of visual inputs (Pavlou and Newham 2013). In addition, older adults may have had less experience with new visual technologies. Both these factors may increase the susceptibility to VIMS. At the same time, an opposing factor comes into play, that overall motion sickness susceptibility to physical motion is known to decline with age (with individual variation) (Paillard et al 2013). Consequently, the relationship of age with VIMS susceptibility warrants investigation in future studies.

The role of biological sex for motion sickness and VIMS has been largely discussed in the past with mixed results. For instance, it has been often stated that females are more susceptible to classical motion sickness (Jokerst et al 1999; Dobie et al 2001) and VIMS (Flanagan et al 2005; Munafo et al 2017; Curry et al 2020a, b) than males, whereas other studies failed to find this sex-related difference (see reviews in Lawson 2014; Lawson et al 2021). In the present study, males appeared slightly more susceptible overall for VIMS, albeit this effect was small and our findings do not suggest strong differences between males and females with regards to VIMS susceptibility. To place this in context, recent reviews have suggested that any increased motion susceptibility in females is a relatively small effect with many contradictory or negative reports especially for VIMS (Lawson 2014; Saredakis et al 2020; Lawson et al 2021). Finally, the very small correlations observed for personality factors with VIMSSQ would further support the conclusion that they are unlikely to be important determinants of motion sickness susceptibility (Reason and Brand 1975).

### General inter-relationships

Factor analysis revealed a single factor loading for the variables VIMSSQ, MSSQ, Migraine, VIC, SWID, and Syncope. This implies the existence of a single underlying latent variable encompassing VIMS together with motion sickness susceptibility, migraine, dizziness, and autonomic reactivity exemplified by syncope. This is similar to findings in large surveys of the general population and patients experiencing vestibular disorders which produce vertigo (Golding and Patel 2017). It has been proposed that there is an underlying set of risk factors which distribute with increasing strength throughout the general population up into what is then termed the ‘clinical population’ for vestibular related disorders such as Visual Vertigo (Peverall and Golding 2017) and Persistent Perceptual Postural Dizziness (PPPD) (Bronstein et al 2020; Powell et al 2020). Further support for this comes from a recent investigation of the VIMSSQ relationship with PPPD. This showed that the VIMSSQ predicted PPPD severity scores (Staab et al 2017; Yagi et al 2019) by *r* = 0.57 and revealed a single factor loading PPPD, VIMSSQ, Migraine, MSSQ, SWID and Syncope, again suggesting a single latent variable of sensitivity (Golding and Jahanara 2022).

### Limitations and future directions

The present study had a number of limitations. Although the age range was wide, the participants were all adults and less is known about the development of VIMS susceptibility in younger people. VIMS susceptibility in children may show stronger age relationships, for instance, a steep rise in susceptibility from very early ages with a possible peak around 8 to 9 years is possible (but see Chang et al. 2021). By analogy, Henriques et al (2014) investigated motion susceptibility in children using an adapted version of the MSSQ: following this logic, an adapted version of the VIMSSQ could be employed as well. Another limitation was possible sampling bias for more susceptible participants. This was implied by the higher than expected MSSQ scores for which good normative data are available. But correction for estimated VIMSSQ norm was possible (see Results) and this provided an estimated normative value for the VIMSSQ.

Another limitation of the present study is that we applied a correlational approach. Although correlation may be consistent with causation, correlational studies cannot, in principle, provide insight into causality. Also, note that this paper does not consider or test the various theories of motion sickness aetiology in detail. We earlier mentioned sensory conflict as a contributor to motion sickness (Reason and Brand 1975), but it is worth noting that other theories concerning the aetiology of motion sickness include postural instability (Stoffregen and Riccio 1988) and other aspects (see Lackner 2014; Golding 2016, for overviews).

Although our study investigated a broad range of traits related to VIMS susceptibility, other potentially relevant factors remain to be assessed in the future. For instance, physical fitness and physical activities were not investigated here but have been suggested as potentially contributing factors to VIMS and classic motion sickness, albeit with mixed findings. For example, it has been shown that aerobic fitness training increased susceptibility to motion sickness (Cheung et al 1990). By contrast, exactly the opposite effects have been observed in dancers and figure skaters, who showed reduced motion sickness susceptibility which may be related to their training (Tanguy et al. 2008; Nigmatulina et al. 2015). Furthermore, it is possible that VIMS and motion sickness susceptibility is related to individual differences in motor control, motor skill, and/or motor learning, and it has been conjectured that it might also be related to biological sex (Munafo et al 2017; Curry et al 2020a, b). In general, the situation with these variables is complicated by the fact that they can have both the properties of a state (i.e. that they can be trained) and a trait (i.e. the effects of this may be enduring), analogous to habituation (see Introduction), which can acquire the properties of a trait if retained over a prolonged term (Benson 2002).

## Conclusion

In conclusion, the results of this study showed that the short version of the VIMSSQ provided a quick and easy to use window into the VIMS susceptibility of a large sample of the population. The set of correlates and predictors for susceptibility to VIMS had similarity with those often observed in patients with vestibular disorders. Based on these correlational results, we suggest the existence of continuum of underlying risk factors for sensitivity, from healthy population to patients with visual vertigo and perhaps Persistent Postural-Perceptual Dizziness.

## Declarations

### Data availability

The datasets generated during and/or analysed during the current study are available from the corresponding author on reasonable request.
